# Supplementary material for: A Pilot Study on the Effects of Transcranial Direct Current Stimulation on Brain Rhythms and Entropy during Self-Paced Finger Movement using the Epoc Helmet
Source: Front Hum Neurosci. 2017 Apr 25;11:201. doi: 10.3389/fnhum.2017.00201 (PMC5408787; doi:10.3389/fnhum.2017.00201)

CL ratio on complete EEG signal for electrodes: P7-P8

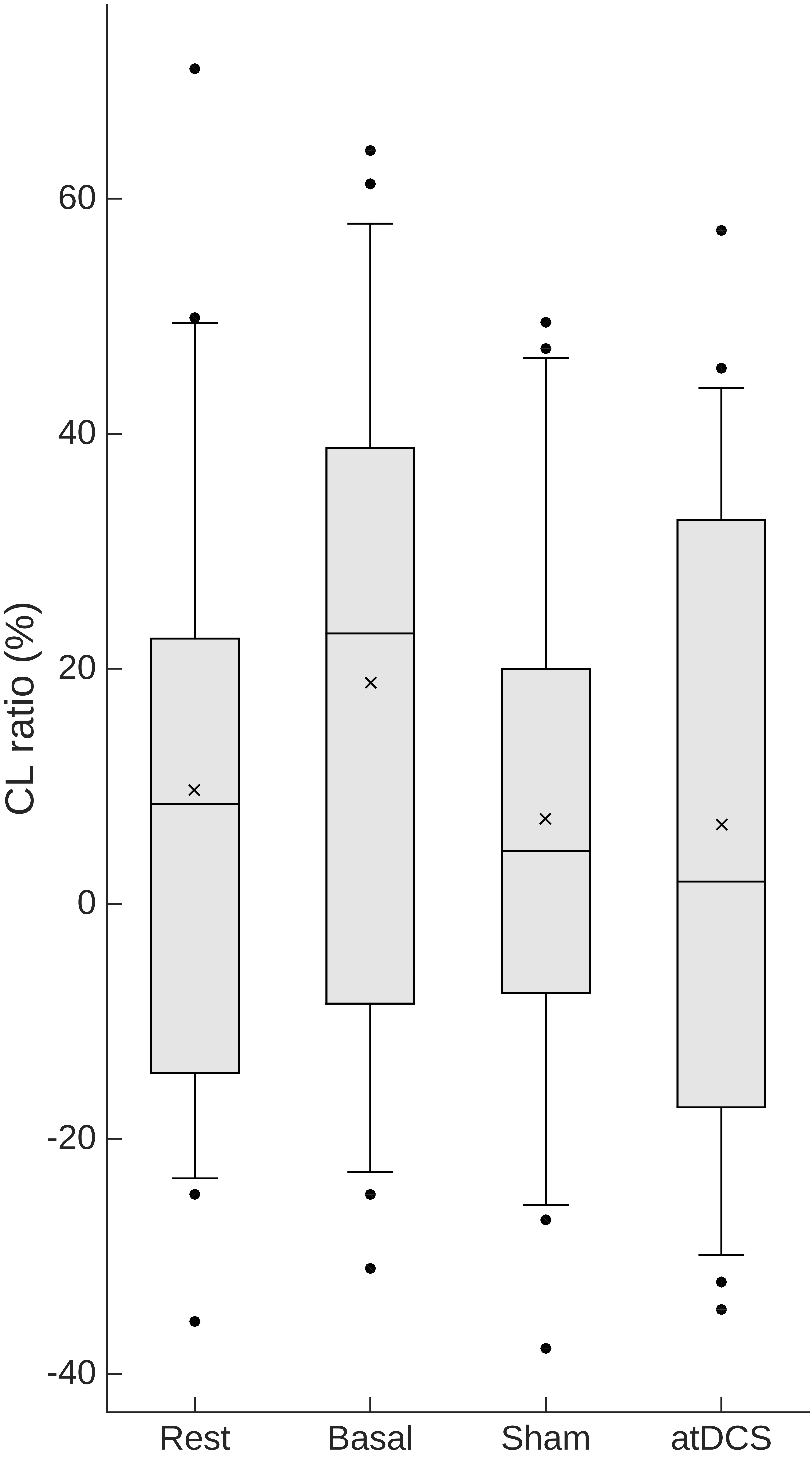

Supplement: Supplementary file 1 [file Data_Sheet_1.zip › Complementary_results/CL-Ratio_Complete_EEG/CL-Ratio_complete-EEG_P7-P8.pdf]
